# The complete chloroplast genome of *Phyllanthus acidus* (L.) Skeels (Phyllanthaceae)

**DOI:** 10.12688/f1000research.140134.1

**Published:** 2023-08-31

**Authors:** Hoang Danh Nguyen, Thi Diem Quynh Nguyen, Minh Thiet Vu, Hoang Dang Khoa Do

**Affiliations:** 1 NTT Hi-Tech Institute, Nguyen Tat Thanh University, Ho Chi Minh City, Vietnam; 2 Faculty of Nursing and Medical Technology, University of Medicine and Pharmacy at Ho Chi Minh City, Ho Chi Minh City, Vietnam

**Keywords:** Malpighiales, Phyllanthaceae, phylogenetic relationships, plastome, star gooseberry

## Abstract

*Phyllanthus acidus* (L.) Skeels (Phyllanthaceae) is a potential medicinal plant recognized for its sour and tart tasted fruits. In this study, the chloroplast genome of *P. acidus* was sequenced, assembled, and characterized. The chloroplast genome size was 156,331 bp and the overall GC content was 36.9%. Additionally, the chloroplast genome had a quadripartite structure consisting of a large single copy (LSC; 85,807 bp in length; GC content: 34.6%), a small single copy (SSC; 19,262 bp in length; GC content: 30.6%), and two inverted repeat regions (IR; 25,631 bp in length; GC content: 43.1%). A total of 113 unique genes were annotated in the chloroplast genome, comprising 79 protein-coding genes, 30 tRNAs, and four rRNAs. The phylogenetic analysis based on 79 protein-coding genes revealed the paraphyly of the *Phyllanthus* genus. These findings provided additional genetic information for further research on *P. acidus* and the cp genome in the Phyllanthaceae family.

## Introduction

*Phyllanthus acidus* (L.) Skeels, also known as star gooseberry, belongs to the Phyllanthaceae family and commonly distributes in the wet tropical regions, including South/ Southeast Asia, Central Africa, the Caribbean region, and Central/South America ( POWO 2022). *P. acidus* has been traditionally used to treat various diseases, including inflammation, gastrointestinal problems, rheumatism, bronchitis, Alzheimer, and hepatic diseases ( Jain *et al*. 2011; Chakraborty *et al*. 2012; Srirama *et al*. 2012; Uddin *et al*. 2016). The leaves and roots of *P. acidus* also possess antidotal properties against viper venom ( Jayvir 1998). Moreover, *P. acidus* exhibited the potential for alleviating hypertension ( Leeya *et al*. 2010).

The chloroplast (cp) genome is extremely effective at inferring phylogeny since it is predominantly maternally inherited, has a conversed structure and gene content, and has a slow mutation frequency ( Palmer *et al*. 1988). Additionally, the cp genomes provide essential data for conducting studies on population genetics, molecular identification, and genetic engineering ( Powell *et al*. 1995; Daniell *et al*. 2016; Cao *et al*. 2022). In the current study, the characteristics of the *P. acidus* cp genome and its phylogenetic implication were explored to gain more information about the evolution and phylogenetic relationships within the Phyllanthaceae family and closely related taxa.

## Methods

### Sample collection

The *P. acidus* sample (young branches with leaves) was collected from Can Tho, Viet Nam (9°56′55.7″N, 105°30′16.0″E) and labeled with voucher number: NTT-2022.12.CR (contact person: Dr. Do Hoang Dang Khoa, dhdkhoa@ntt.edu.vn). It was deposited at the NTT Hi-tech Institute, Nguyen Tat Thanh University. No specific permit was required to collect and study the species in Vietnam. The leaf sample was dried with silica gel and stored in a -80°C freezer until conducting DNA extraction.

### Data collection and analysis

The total genomic DNA extraction from the dried leaves was carried out using the Cetyltrimethylammonium bromide (CTAB) protocol ( Doyle & Doyle 1987). The quality of genomic DNA samples was checked using gel electrophoresis and NanoDrop One ^C^ Spectrophotometer. The DNA samples that showed a clear band on agarose gel and had a 260/280 ratio between 1,8-2 and a 260/230 ratio between 2.0-2.2 were selected for conducting the next-generation sequencing step. Subsequently, the library was prepared with the NEBNext Ultra II DNA Library Prep Kit for Illumina (NEB, USA). The library was sequenced using Illumina Miseq platform to generate paired-end reads of 150 bp (Ktest Science Co. Ltd., Vietnam). The raw reads were qualified and filtered low-quality reads (Q score < 20 and length < 100 bp) and reads containing primers or adapters using FastQC v0.12.1 and Trimmomatic v0.39 programs ( Andrews 2010; Bolger *et al*. 2014). For the assembly of cp genome, the NOVOPlasty v4.3.1 program was used ( Dierckxsens *et al*. 2017). Preliminary annotation was conducted by Geseq with default parameters ( Tillich *et al*. 2017). The complete annotation genome was illustrated using OrganellarGenomeDRAW v1.3.1 ( Greiner *et al*. 2019). All 79 protein-coding regions in the cp genomes of *P. acidus* and 16 related taxa from the Phyllanthaceae were extracted and aligned for phylogenetic analysis using MUSCLE v5 program ( Edgar 2004). The chloroplast genome of *Acalypha hispida* (Euphorbiaceae; Genbank accession no. NC_070339) was selected as an outgroup. A maximum likelihood phylogenetic tree was reconstructed using IQTREE with 1000 bootstrap replicates and GTRGAMMA substitution model ( Nguyen *et al*. 2015).

## Results

**Figure 1.**
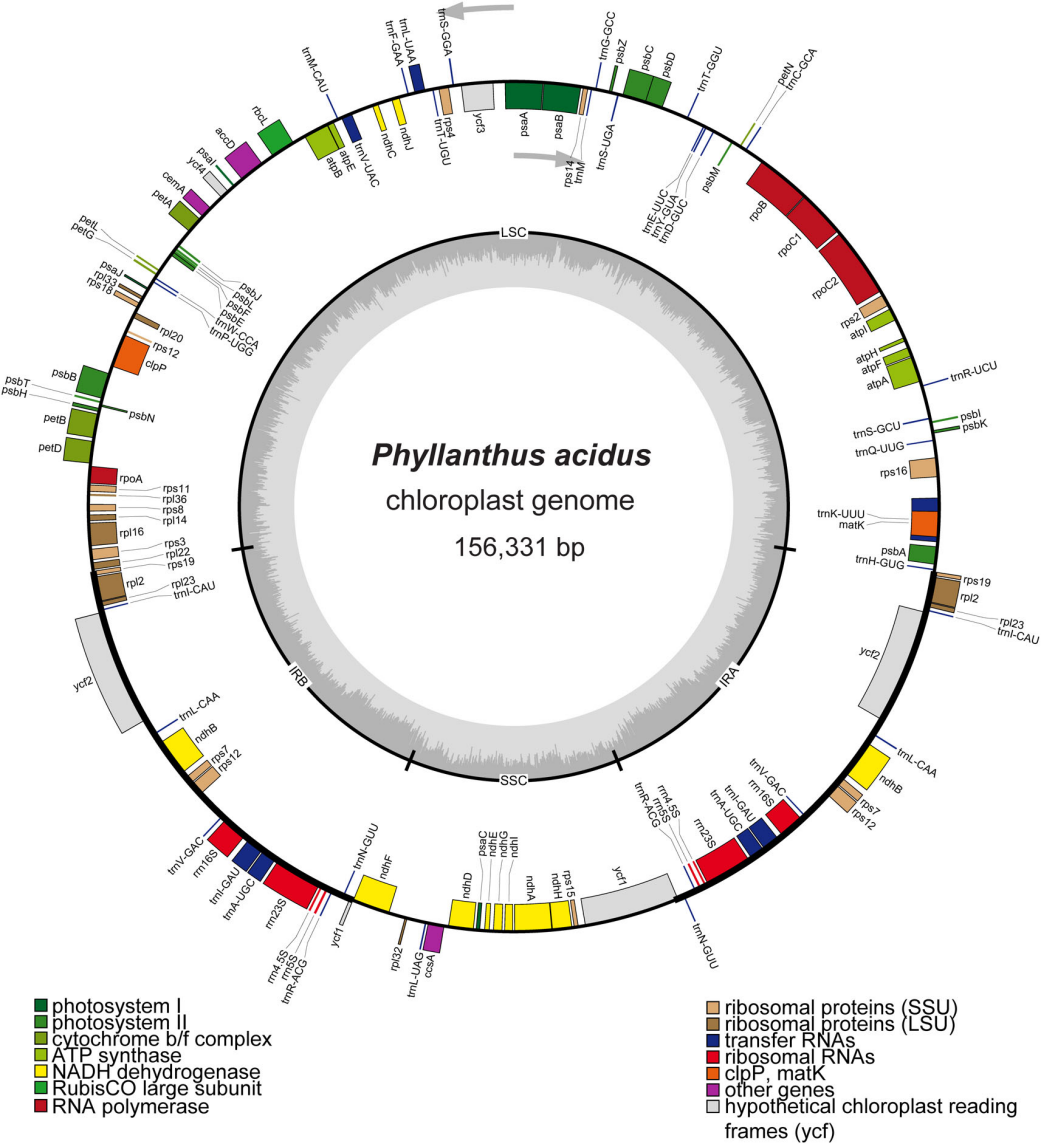
Map of the cp genome of *Phyllanthus acidus.* Genes located inside the circle are transcribed in a clockwise direction, while genes outside the circle are transcribed counterclockwise. The inner circle depicted in dark gray that represents the GC content, while the light-gray circle represents the AT content of the genome. LSC: large single copy; SSC: small single copy; IRA/IRB: inverted repeat regions.

**Figure 2.**
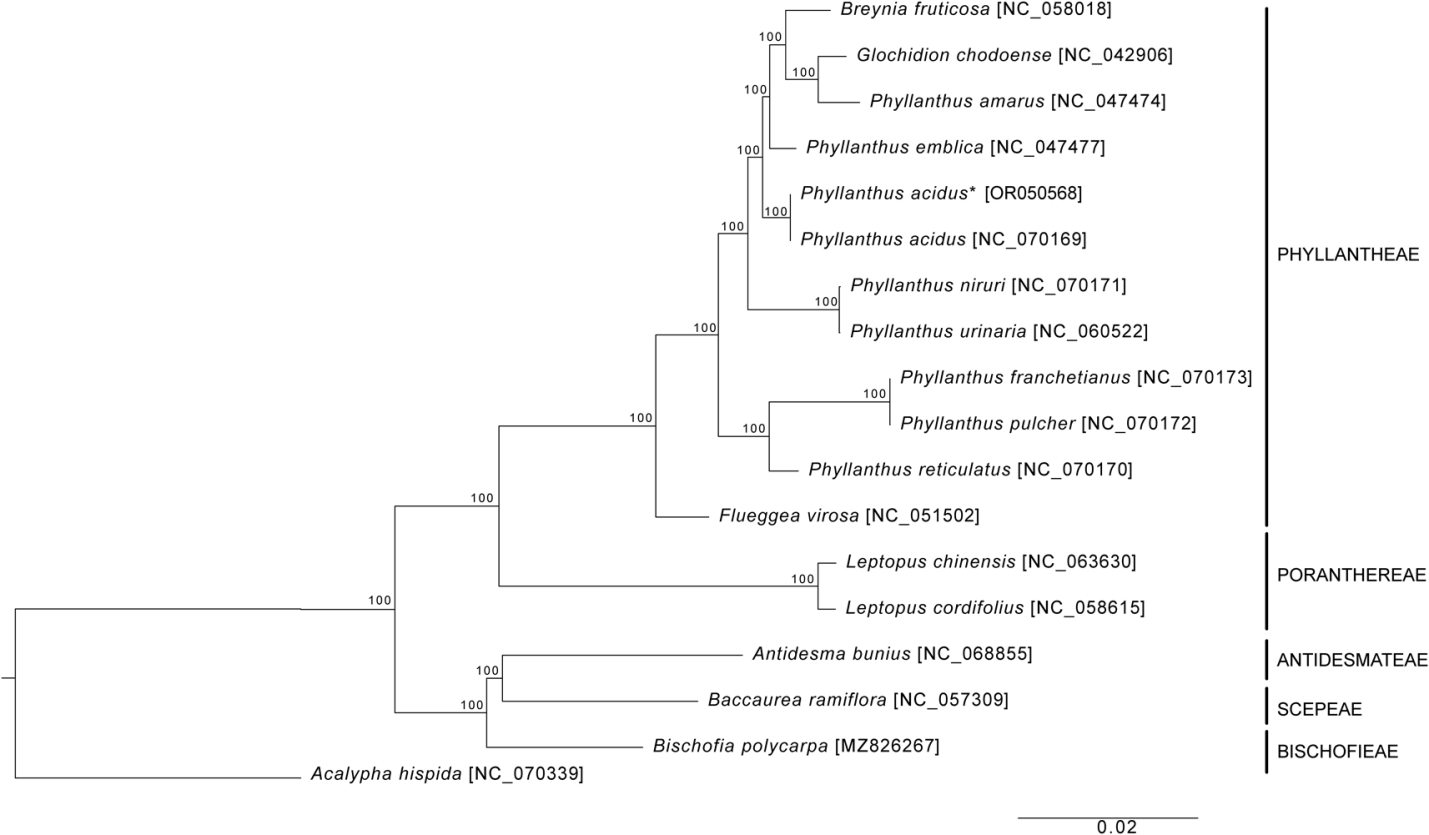
The ML phylogenetic tree of *Phyllanthus acidus* and related species based on the 79 protein-coding sequences of cp genome . The asterisk indicates *P. acidus* sequenced in this study. The numbers next to each node are bootstrap values.

Approximately 349.8 MB of clean reads were obtained and used for completing the cp genome of *P. acidus.* The assembly process utilized 1,166,034 paired-end reads, resulting in an average coverage depth of 2,234.3X ( Nguyen, Nguyen, Do, and Vu, 2023; Nguyen, Do, and Vu, 2023) The quadripartite cp genome of *P. acidus* (GenBank accession number OR050568) had a length of 156,331 bp and consisted of an LSC region of 85,807 bp, a SSC region of 19,262 bp, and a pair of IR regions of 25,631 bp ( Figure 1). The overall GC content of the genome was 36.9%, and the GC content of the LSC, SSC, and IR regions were 34.6%, 30.6%, and 43.1%, respectively. The cp genome of *P. acidus* contained a total of 130 genes, including 85 protein-coding regions, 37 tRNA genes, and eight rRNA genes ( Table 1). Among 85 protein-coding genes, 17 genes contained intron, of which *ycf3* and *clpP* contained two introns. In IR regions, a total of 19 genes were duplicated, including eight protein-coding regions (i.e., *rps19, rpl2, rpl23, ycf1, ycf2, ndhB*, *rps12*, and *rps7*), seven tRNAs ( *trnI_CAU, trnL_CAA, trnV_GAC, trnI_GAU, trnA_UGC, trnR_ACG*, and *trnN_GUU*), and four rRNAs ( *rrn16S, rrn23S, rrn4.5S*, and *rrn5S*). Notably, *rps19* and *ycf1* duplications were incomplete. The phylogenetic analysis revealed a paraphyly of *Phyllanthus* species *,* in which *Breynia futicosa* and *Glochidion chodoense* formed a clade with *Phyllanthus amarus* ( Figure 2). Therefore, more genomic data and samples of Phyllanthaceae species are required for further phylogenetic studies.

**Table 1.** List of genes in the chloroplast genome of *Phyllanthus acidus.*

Groups of genes	Name of genes
Ribosomal RNAs	*rrn4.5(2x), rrn5(2x), rrn16(2x), rrn23(2x)*
Transfer RNAs	*trnA_UGC* * *(2x), trnC_GCA, trnD_GUC, trnE_UUC, trnF_GAA, trnG_UCC* * *, trnG_GCC, trnH_GUG, trnI_GAU* * *(2x), trnK_UUU* * *, trnL_CAA(2x), trnL_UAA* * *, trnL_UAG, trnfM_CAU, trnM_CAU(2x), trnM_CAU, trnN_GUU(2x), trnP_UGG, trnQ_UUG, trnR_ACG(2x), trnR_UCU, trnS_GCU, trnS_GGA, trnS_UGA, trnT_GGU, trnT_UGU, trnV_GAC(2x), trnV_UAC* * *, trnW_CCA, trnY_GUA*
Photosystem I	*psaA, psaB, psaC, psaI, psaJ*
Photosystem II	*psbA, psbB, psbC, psbD, psbE, psbF, psbH, psbI, psbJ, psbK, psbL, psbM, psbN, psbT, psbZ*
Cytochrome	*petA, petB* * *, petD* * *, petG, petL, petN*
ATP synthases	*atpA, atpB, atpE, atpF* * *, atpH, atpI*
Large unit of Rubisco	*rbcL*
NADH dehydrogenase	*ndhA* * *, ndhB* * *(2x), ndhC, ndhD, ndhE, ndhF, ndhG, ndhH, ndhI, ndhJ, ndhK*
ATP-dependent protease subunit P	*clpP* *
Envelop membrane protein	*cemA*
Large units of ribosome	*rpl2* * *(2x), rpl14, rpl16* * *, rpl20, rpl22, rpl23(2x), rpl32, rpl33, rpl36*
Small units of ribosome	*rps2, rps3, rps4, rps7(2x), rps8, rps11, rps12* * *(2x), rps14, rps15, rps16* * *, rps18, rps19(2xa)*
RNA polymerase	*rpoA, rpoB, rpoC1* * *, rpoC2*
Initiation factor	*infA*
Other genes	*accD, ccsA, matK*
Hypothetical proteins and conserved reading frames	*ycf1(2xa), ycf2(2x), ycf3* * *, ycf4*

* Gene with introns; 2x – duplicated gene in IR region; 2xa – incomplete duplicated gene in IR region.

## Data Availability

NCBI Short Read Archive (SRA): DNA-seq of *Phyllanthus acidus.* Accession number SRR24772537; https://www.ncbi.nlm.nih.gov/sra/SRR24772537 ( Nguyen, Nguyen, Do, and Vu, 2023). NCBI Assembly database: *Phyllanthus acidus* chloroplast, complete genome. Accession number OR050568; https://www.ncbi.nlm.nih.gov/nuccore/OR050568 ( Nguyen, Do, and Vu, 2023).
