# Supplementary material for: The complete chloroplast genome of Phyllanthus acidus (L.) Skeels (Phyllanthaceae)
Source: F1000Res. 2025 Nov 21;12:1059. Originally published 2023 Aug 31. [Version 3] doi: 10.12688/f1000research.140134.3 (PMC12686778; doi:10.12688/f1000research.140134.3)
Supplement: Peer review history

# Reviewer report: Reviewer response for version 3

**DOI:** 10.5256/f1000research.190830.r439860

**Published:** 2026-01-07

**Authors:** Sunil Kumar Sahu

**Affiliations:** 1 BGI-Shenzhen, Shenzhen, China

This Genome Note is technically sound and presents a well-covered *Phyllanthus acidus* plastome, accompanied by open data and a straightforward phylogenetic analysis that agrees with previous plastome-based inferences for Phyllanthaceae. The study has clear potential value, both because *P. acidus* is medicinally important and because the clade itself is taxonomically challenging. That said, several aspects still need clarification or correction before the manuscript can be considered fully robust. In particular, the rationale for generating a new plastome despite the existence of an earlier *P. acidus* assembly needs to be explicitly stated, along with any improvements your dataset contributes. My specific comments and questions are below:

 • What is the explicit justification for re-sequencing *P. acidus* given the available GenBank plastome (N C_070169)? Please indicate whether your assembly provides concrete improvements or corrections (for example, IR boundary resolution, the extent of *ycf1*/ *rps19* pseudogenization, or annotation differences).

 • The manuscript reports inconsistent gene counts: the Abstract lists 113 unique genes (79 PCGs, 30 tRNAs, 4 rRNAs) whereas the Results and Table 1 suggest 130 total genes (85 PCGs, 37 tRNAs, 8 rRNAs). Please clarify which numbers refer to unique genes versus totals including IR duplicates, and ensure Table 1 reflects accurate gene names and counts.

 • Table 1 contains several typographical issues (for example, Cyrillic characters in *rpo*-genes, duplicated *trnM-CAU*, and the inclusion of “ *rps1*”). Please correct these using your GeSeq output, and explicitly indicate that *rps12* is a trans‑spliced gene.

 • Please specify the seed sequence and parameters used in NOVOPlasty, and describe how you validated genome circularization and IR/SC junction accuracy (for example, via read‑mapping coverage, junction‑spanning reads, or targeted PCR).

 • For the phylogenetic analyses, please report the exact models used in IQ‑TREE and MrBayes, using each program’s proper nomenclature. Indicate whether you performed model selection (e.g., ModelFinder), partitioning (gene or codon level), alignment trimming or masking, or any saturation testing.

 • If you have compared your plastome directly to NC_070169 (e.g., pairwise divergence, structural differences, annotation discrepancies), please summarize the key findings. If not, a brief comparison would strengthen the manuscript.

 Overall, the work is promising and close to solid, but addressing these issues will substantially improve clarity, reproducibility, and the manuscript’s contribution to plastome resources for *Phyllanthus*.

Are the datasets clearly presented in a usable and accessible format, and the assembly and annotation available in an appropriate subject-specific repository?

Yes

Are sufficient details of the sequencing and extraction, software used, and materials provided to allow replication by others?

Yes

Are the rationale for sequencing the genome and the species significance clearly described?

Yes

Are the protocols appropriate and is the work technically sound?

Yes

Reviewer Expertise:

Plant genomics and evolution

I confirm that I have read this submission and believe that I have an appropriate level of expertise to confirm that it is of an acceptable scientific standard, however I have significant reservations, as outlined above.

# Reviewer report: Reviewer response for version 3

**DOI:** 10.5256/f1000research.190830.r435864

**Published:** 2025-12-11

**Authors:** Yoshinori Fukasawa

**Affiliations:** 1 Center for Bioscience Research and Education Utsunomiya University, Tochigi, Japan

I agree with the other reviewers. The manuscript clearly describes the importance of *P. acidus* as a species and provides a technically sound chloroplast assembly with appropriate sequencing and annotation procedures. These strengths are appreciated.

 However, there is already an existing *P. acidus* chloroplast genome available in NCBI, and the Genome Note guidelines require authors to explain “ *why and how the genomes were sequenced*.” Simply removing the earlier incorrect statement (“has not been studied”) or adding comments on the species’ economic or medicinal value does not address the core issue raised by the reviewer. The manuscript should explicitly clarify the rationale for resequencing the chloroplast genome despite the presence of a previously published assembly—e.g., whether the new (meta)data provide validation, improved quality, structural corrections, or broader comparative relevance.

 Minor technical corrections

 Additionally, several tool names should be standardized (e.g., GeSeq, IQ-TREE), and the substitution model should match the nomenclature actually used in IQ-TREE or MrBayes, as “GTRGAMMA” is not a conventional model designation in those programs.

Are the datasets clearly presented in a usable and accessible format, and the assembly and annotation available in an appropriate subject-specific repository?

Yes

Are sufficient details of the sequencing and extraction, software used, and materials provided to allow replication by others?

Yes

Are the rationale for sequencing the genome and the species significance clearly described?

Partly

Are the protocols appropriate and is the work technically sound?

Yes

Reviewer Expertise:

Bioinformatics, genome assembly, and computational genomics.

I confirm that I have read this submission and believe that I have an appropriate level of expertise to confirm that it is of an acceptable scientific standard, however I have significant reservations, as outlined above.

# Reviewer report: Reviewer response for version 3

**DOI:** 10.5256/f1000research.190830.r434949

**Published:** 2025-12-08

**Authors:** Qiang Li

**Affiliations:** 1 Anhui Normal University, Wuhu, Anhui, China; 2 Nanjing Forestry University, Nanjing, China

I have finished reading the revised manuscript. The article reads very well, and I have no further comments.

Are the datasets clearly presented in a usable and accessible format, and the assembly and annotation available in an appropriate subject-specific repository?

Yes

Are sufficient details of the sequencing and extraction, software used, and materials provided to allow replication by others?

Yes

Are the rationale for sequencing the genome and the species significance clearly described?

Partly

Are the protocols appropriate and is the work technically sound?

Yes

Reviewer Expertise:

NA

I confirm that I have read this submission and believe that I have an appropriate level of expertise to confirm that it is of an acceptable scientific standard.

# Reviewer report: Reviewer response for version 2

**DOI:** 10.5256/f1000research.189635.r426288

**Published:** 2025-11-03

**Authors:** Shiou Yih Lee

**Affiliations:** 1 INTI International University, Nilai, Malaysia

The manuscript describes on the characterisation of the chloroplast genome sequence of Phyllanthus acidus. My comments are as follows:

 1. There is one chloroplast genome sequence of P. acidus in NCBI. The problem statement and objective of this study are not very relevant.

 2. How did the authors extract the protein-coding sequences from the chloroplast genome sequence for phylogenetic analysis?

 3. As there is another record of P. acidus in NCBI GenBank, no discussion or comparison was conducted between that and the one from this study. The placement of P. acidus of this study in the phylogenetic tree was not described in the Results. There is no discussion on the findings from the phylogenetic analysis.

Are the datasets clearly presented in a usable and accessible format, and the assembly and annotation available in an appropriate subject-specific repository?

Yes

Are sufficient details of the sequencing and extraction, software used, and materials provided to allow replication by others?

Yes

Are the rationale for sequencing the genome and the species significance clearly described?

Yes

Are the protocols appropriate and is the work technically sound?

Yes

Reviewer Expertise:

forestry, molecular phylogenetics and evolutionary

I confirm that I have read this submission and believe that I have an appropriate level of expertise to confirm that it is of an acceptable scientific standard, however I have significant reservations, as outlined above.

# Response

**Published:** 2025-11-04

**Authors:** Hoang Dang Khoa Do

**Affiliations:** Nguyen Tat Thanh University NTT Hi-Tech Institute, Ho Chi Minh City, Ho Chi Minh, Vietnam

Dear Reviewer,

 Thank you very much for your comments and suggestions.

 We revised the manuscript based on your suggestions.

 Please check our responses as mentioned below.

 The manuscript describes on the characterisation of the chloroplast genome sequence of *Phyllanthus acidus*. My comments are as follows:

 1. There is one chloroplast genome sequence of *P. acidus* in NCBI. The problem statement and objective of this study are not very relevant.

**Response:** Thank you very much for your comment. In the original version, we did not mention as "The current study explored the characteristics of the *P. acidus* cp genome which has not been studied" which was added in the revised version. We are so sorry for the wrong information. We revised this information in the newest version of our manuscript.

 2. How did the authors extract the protein-coding sequences from the chloroplast genome sequence for phylogenetic analysis?

**Response:** Thank you very much for your question. We added the program for extracting protein-coding genes which is Geneious Prime v2023.0 in the revised manuscript.

 3. As there is another record of *P. acidus* in NCBI GenBank, no discussion or comparison was conducted between that and the one from this study. The placement of *P. acidus* of this study in the phylogenetic tree was not described in the Results. There is no discussion on the findings from the phylogenetic analysis.

**Response:** Thank you very much for your comment. We submitted our manuscript as a Genome Note type which requires "brief descriptions of genome sequences that include details of why and how the genomes were sequenced; they do not include any analyses or conclusions.". Therefore, we focused on description of our target genome and did not do comparative genomic analyses. In addition, in Genome Note type, the regulation does not allow a Discussion part in the manuscript ( https://f1000research.com/for-authors/article-guidelines/genome-notes). Therefore, we did not discuss in depth about phylogenetic analysis in our manuscript. We added more details about phylogenetic relationship between *Phyllanthus acidus* in the main text.

 Thank you.

# Reviewer report: Reviewer response for version 2

**DOI:** 10.5256/f1000research.189635.r426285

**Published:** 2025-11-03

**Authors:** Qiang Li

**Affiliations:** 1 Anhui Normal University, Wuhu, Anhui, China; 2 Nanjing Forestry University, Nanjing, China

*Phyllanthus* is one of the taxonomic difficulties genera of Phyllanthaceae. It contains approximately 800 species, mainly distributed in the tropical and subtropical regions of the world, and a few are in the northern temperate zone. Previous studies have demonstrated that *Phyllanthus* is a paraphyletic group. Several solutions have been proposed for dividing *Phyllanthus* species. Some researchers classified previously established classifications into *Phyllanthus* sensu lato, such as (Kathriarachchi et al. 2006), who placed *Breynia, Glochidion *and *Synostemon *into *Phyllanthus*. Others split *Phyllanthus *into several monophyletic genera. However, these taxonomic evidence lack support from genomics. Therefore, we need to obtain more genomic data of this genus to better resolve the taxonomic controversies within it. I believe this is also the most important significance of this manuscript reporting the chloroplast genome of *Phyllanthus acidus* .

 The primary objective of this manuscript is to assemble and provide a basic description of the *Phyllanthus acidus* genome. The methods and content are straightforward and easy to follow. I have two main concerns that I hope the authors will address to enhance the quality of the manuscript.

 [1] I agree with the previous reviewer's opinion that the introduction lacks the significance of studying the chloroplast genome of this species. I suggest that the authors could add a concise introduction. For example, there is controversy over the broad and narrow senses of the genus *Phyllanthus*, and this molecular phylogenetic debate lacks evidence from genomics. Therefore, the genome sequenced in this study lays the foundation for resolving the taxonomic controversy of the genus *Phyllanthus *in the future.

 [2] OrganellarGenomeDRAW requires a reference genome for annotating chloroplast genomes, and the authors should specify which species' chloroplast genome was used as the reference.

Are the datasets clearly presented in a usable and accessible format, and the assembly and annotation available in an appropriate subject-specific repository?

Yes

Are sufficient details of the sequencing and extraction, software used, and materials provided to allow replication by others?

Yes

Are the rationale for sequencing the genome and the species significance clearly described?

Partly

Are the protocols appropriate and is the work technically sound?

Yes

Reviewer Expertise:

My research areas primarily encompass speciation, phylogenetics, and the conservation of endangered plants. Currently, I mainly utilize chloroplast genomes, mitochondrial genomes, and nuclear genomes to address evolutionary questions.

I confirm that I have read this submission and believe that I have an appropriate level of expertise to confirm that it is of an acceptable scientific standard, however I have significant reservations, as outlined above.

## References

[1] : The complete chloroplast genome of Phyllanthus acidus (L.) Skeels (Phyllanthaceae). *F1000Research* .2025;12: 10.12688/f1000research.140134.2 10.12688/f1000research.140134.2 PMC1268677841378226

# Response

**Published:** 2025-11-03

**Authors:** Hoang Dang Khoa Do

**Affiliations:** Nguyen Tat Thanh University NTT Hi-Tech Institute, Ho Chi Minh City, Ho Chi Minh, Vietnam

Dear Reviewer,

 Thank you very much for your helpful comments.

 We revised the manuscript based on your comments. 

 Please check our response as mentioned below.

 The primary objective of this manuscript is to assemble and provide a basic description of the *Phyllanthus acidus* genome. The methods and content are straightforward and easy to follow. I have two main concerns that I hope the authors will address to enhance the quality of the manuscript.

 [1] I agree with the previous reviewer's opinion that the introduction lacks the significance of studying the chloroplast genome of this species. I suggest that the authors could add a concise introduction. For example, there is controversy over the broad and narrow senses of the genus Phyllanthus, and this molecular phylogenetic debate lacks evidence from genomics. Therefore, the genome sequenced in this study lays the foundation for resolving the taxonomic controversy of the genus *Phyllanthus *in the future.

**Response: **Thank you very much for your suggestion. We revised the second part of the Introduction with an adding of a previous comparative genomic analysis based on complete chloroplast genomes of *Phyllanthus *which indicated a non-monophyletic status of *Phyllanthus *species. In addition, we modified the aims of the current study as "Therefore, in the current study, we explored the characteristics of the *P. acidus cp* genome and its phylogenetic implication to gain more genomic information about the chloroplast genome evolution and to provide more data to resolve controversy of phylogenetic relationships within the *Phyllanthus* genus and closely related taxa in *Phyllanthaceae*.".

 [2] OrganellarGenomeDRAW requires a reference genome for annotating chloroplast genomes, and the authors should specify which species' chloroplast genome was used as the reference.

**Response:** Thank you very much for your suggestion. We checked the paper ( https://doi.org/10.1093/nar/gkz238) and the website ( https://chlorobox.mpimp-golm.mpg.de/OGDraw.html) of OGDRAW and we did not find any regulation to select a reference genome for annotation chloroplast genome before drawing the chloroplast genome map. The input of OGDRAW is an annotated file (a GenBank flat file) of chloroplast genome which included all information of genes. Then OGDRAW will converts annotations in GenBank format to graphical maps.

 For annotating chloroplast genome using GESEQ, there is an option for selecting the reference genome. We selected the default reference set of the Geseq which is "MPI-MP Reference Set chloroplast land plants (CDS + rRNA)". We did not use a reference genome from *Phyllanthus *species because we would like to compare our sequence to the land plant database. The more annotation information we compare, the more accuracy of chloroplast genome annotation we get.

 Thank you.

# Reviewer report: Reviewer response for version 1

**DOI:** 10.5256/f1000research.153464.r202362

**Published:** 2024-01-22

**Authors:** Jessica D. Rey

**Affiliations:** 1 Plant Molecular Phylogenetics Laboratory, Institute of Biology, College of Science, University of the Philippines Manila, Manila, Metro Manila, Philippines

This is a good genome note on the sequencing and assembly of the plastome of *Phyllanthus acidus*. However, the paper lacks enough details on the reasons behind investigating the species (i.e., limited genetic and genomic resources? problems with identification?).

 The protocol on sequencing and assembly is appropriate and is clearly described in the paper. To improve data transparency, I suggest including supplementary data such as depth of coverage/read mapping depth and the list of genes which are hard to annotate (i.e., cis-splicing/trans-splicing genes). In addition, I would also like to suggest the use of at least two different methods of phylogenetic analyses (e.g., Maximum likelihood and Bayesian inference) and to indicate the statistical support values [of the two methods] in the tree.

 Lastly, to improve readability of the paper I would like to recommend checking for grammatical errors.

Are the datasets clearly presented in a usable and accessible format, and the assembly and annotation available in an appropriate subject-specific repository?

Yes

Are sufficient details of the sequencing and extraction, software used, and materials provided to allow replication by others?

Yes

Are the rationale for sequencing the genome and the species significance clearly described?

Partly

Are the protocols appropriate and is the work technically sound?

Yes

Reviewer Expertise:

Molecular characterization of different plant species i.e. Philippine endemic medicinal plants, strawberry varieties planted in the Philippines, genetics of disease resistance cultivated rice, soilless culture of leafy and fruiting vegetables

I confirm that I have read this submission and believe that I have an appropriate level of expertise to confirm that it is of an acceptable scientific standard, however I have significant reservations, as outlined above.

# Response

**Published:** 2025-10-12

**Authors:** Hoang Dang Khoa Do

**Affiliations:** Nguyen Tat Thanh University NTT Hi-Tech Institute, Ho Chi Minh City, Ho Chi Minh, Vietnam

Dear Reviewers,

 Thank you very much for your helpful comments.

 1/ We added the reason for conducting genomic research about *Phyllanthus acidus* in the Introduction as “which has not been studied”.

 2/ We added the information of depth of coverage/read mapping depth and cis-splicing/trans-splicing genes as supplementary files.

 3/ We revised the phylogenetic tree with the Bayesian inference method.

 Best regards.
